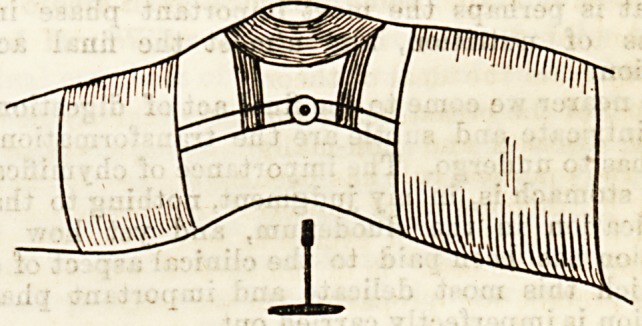# Treatment of Stiff Joints

**Published:** 1893-07-22

**Authors:** 


### ROYAL ORTHOPÆDIC HOSPITAL.

### TREATMENT OF STIFF JOINTS.

It is obvious that these must be treated according to the cause that produces them. If the cause of the stiffness is inflammation outside the joint, it is kept at rest, or otherwise treated in such way as appears best to subdue the inflammation. When that has subsided, massage and manipulation are used in order to restore the freedom of the movement. In this and all other forms of stiff joint, the rubbing is made with some form of soap liniment. At first the joint is handled very gently, and then as the tenderness disappears, more and more force is used. It is considered that the liniment used is a matter of little consequence, and that the more the joint is rubbed, the better the result that will follow.

If the stiffness is due to adhesions inside the joint, massage is used as in the previous cases, but in addition the joint is passively worked in order to break down any adhesions that may have formed between the joint surfaces. The method of conducting this manipulation is a matter of considerable consequence. It is best performed under chloroform, because it is generally painful, and further, without chloroform it is impossible to estimate the actual amount of fixation and the range of movement that has been obtained. In working joints under chloroform, great care is taken that the amount of force shall be as small as possible, that it be exerted rather so as to bend than to extend the joints, and that in any case it be applied so that no strain be put on vessels, nerves, or other important structures in the neighbourhood. This precaution is necessary, since great damage has been caused by the use of too great force, or its application in improper directions. Of course, if free movement can be obtained by this working and massage, no other treatment is required; but in a good many cases the adhesions are too strong to be broken by any force that can be safely exerted, or the stiffness is not due to them, but to contraction and thickening of bands in ligaments, tendons, or other structures outside the joint. If the tendons be tight they are divided, and the other tissues are acted upon by means of a powerful screw instrument.

This, as shewn in the diagram, consists in its simplest form of metal bands above and below the joint, fastened together by iron rods down the sides of the limb, which are hinged opposite the joint, the hinge being regulated by a rack and pinion mechanism. A cap over the convexity of the joint completes the instrument. By its use a steady and continuous pressure can be exerted in such a way as to straighten the limb. The instrument requires to be regulated in order to prevent the formation of pressure sores, and sometimes the pressure has to be diminished because of pain or inflammation of the joint.

Movement obtained in the manner above described can generally be maintained or increased by passive movements, or by active exertion on the part of the patient. At the same time, the rubbing with liniment is persevered in. If the stiffness is due to bony anchylosis of the joint, it is obvious that the treatment described above can be of little value, and that if any change is to be made it must be by means of some cutting operation. Before performing such an operation the case is carefully considered. If it be the leg that is affected, a straight stiff joint is much more useful than a weak moveable one. Further, it is generally found impossible to obtain by operation a strong moveable joint in the leg. The general opinion is, therefore that operations should not be performed for anchylosis of the joints of the leg, if it be straight or nearly so, and that when performed, the object should be to obtain a straight strong leg with a fixed joint, so that it can be walked on.

If the arm be affected the case is somewhat different. Movement is with many people a greater necessity in the arm than strength, and therefore an operation is sometimes performed simply in order to get a moveable joint. Such an operation is considered more advisable at the elbow than at the other joints, seldom being advisable in the wrist, strength being of considerable consequence there, and a good moveable joint being difficult or impossible to obtain by operation.

## Figures and Tables

**Figure f1:**